# Invasive maxillary aspergillosis in a patient with systemic lupus erythematosus: Case report

**DOI:** 10.1016/j.amsu.2020.08.031

**Published:** 2020-09-01

**Authors:** Ishandono Dachlan, Aditya Wicaksana, Aditya Rifqi Fauzi, Siti Isya Wahdini, Nurardhilah Vityadewi, Muhammad Rosadi Seswandhana, Muhammad Bakhrul Lutfianto, Franciscus Wihan Pradana

**Affiliations:** a Plastic Reconstructive and Aesthetic Surgery Division, Department of Surgery, Faculty of Medicine, Public Health and Nursing/Dr. Sardjito Hospital, Yogyakarta, 55281, Indonesia; b Department of Oral and Maxillofacial Surgery, Faculty of Dentistry/Dr. Sardjito Hospital, Yogyakarta, 55281, Indonesia; c Department of Prosthodontics, Faculty of Dentistry/Dr. Sardjito Hospital, Yogyakarta, 55281, Indonesia

**Keywords:** Invasive aspergillosis, Systemic lupus erythematosus, Palatal defect, Maxillary defect, Plastic reconstructive surgery, IA, Invasive aspergillosis, SLE, systemic lupus erythematosus, anti-dsDNA, anti-double stranded DNA, ANA, antinuclear antibodies, ENT, ear, nose, and throat, CT scan, computed tomography, PAS, Periodic Acid-Schiff, MRI, magnetic resonance imaging, GMS, Grocott-Gomori's Methenamine Silver, FRS, fungal rhinosinusitis

## Abstract

**Introduction:**

Invasive aspergillosis (IA) is a fungal infection caused by Aspergillus species (spp.). Aspergillosis is the most common source of opportunistic fungal infection in humans. IA can cause serious complications related to high morbidity and mortality in immunocompromised patients.

**Presentation of case:**

We report a case of a 22-year-old female with a chief complaint of having a hole in the roof of her mouth. She was diagnosed with SLE in 2009. She had been consuming oral methylprednisolone ever since. In 2018, she experienced worsened symptoms and was hospitalized. She experienced swelling and bleeding of her gums and some of her teeth becoming loose and falling out, and then developing a hole in the roof of her mouth. Subsequently, she was treated with oral cyclophosphamide, oral mycophenolate sodium, and oral fluconazole. She was asked to stop taking oral methylprednisolone. In 2019, the palate biopsy was performed and showed *Aspergillus* spp. invading the palate. Afterward, the patient was referred to our clinic for defect closure. The patient was operated on for debridement and reconstruction of the defect. There was no recurrence of the defect or complications observed in the follow-up. The patient was satisfied with the surgical results.

**Discussion:**

IA is a destructive and potentially harmful opportunistic fungal infection and treatments with surgical interventions should be well-thought-out in immunocompromised patients.

**Conclusion:**

The management of IA are controlling any underlying diseases and surgical debridement or necrotomy. Generally, antifungal therapy and prompt surgical intervention are successful in managing invasive aspergillosis.

## Highlights

• IA can cause serious complications, especially immunocompromised patients.
• The management can vary from pharmacological therapy to surgical intervention.
• Antifungal therapy and prompt surgical intervention are successful in managing invasive aspergillosis.

## 1 Introduction

Invasive aspergillosis (IA) is a fungal infection caused by the *Aspergillus* species (spp.). Aspergillosis is the most common opportunistic fungal infection in humans, which usually affects the pulmonary tract but can also infect any organ, including the skin, bones, sinuses, cerebral meninges, myocardium, liver, thyroid, and renal tissues [1]. IA can cause serious complications related to high morbidity and mortality in humans, especially immunocompromised patients such as those with systemic lupus erythematosus (SLE) and chronic steroid users [2].

Compared to healthy individuals, patients with autoimmune diseases such as SLE possess a twofold risk of getting opportunistic infections including IA. It may be caused by immunosuppressive drugs but can also be due to the primary immune dysregulation or other autoimmune disease manifestations such as lymphopenia [3].

*Aspergillus* spp. are a type of fungus that lives indoors and outdoors. Only a few of their strains are pathogenic in humans. *Aspergillus fumigatus* and *Aspergillus flavus* are among the most common species causing fungal infection in humans [4].

IA usually appears in the palate or tongue as a painful necrotic destructive lesion and has a yellow or grey slough [5]. Once *Aspergillus* spp. Inoculates the oral epithelium, their hyphae can enter host tissues through the release of toxins, including phthalic acid, aflatoxin, gliotoxin, hemolysin, phospholipases, and several proteases [6]. *Aspergillus* spp. can also disseminate hematogenously resulting in secondary thrombosis and hemorrhage, leading to tissue necrosis and prompt systemic infection and inflammation [7].

Fungal rhinosinusitis (FRS) is classified into invasive and non-invasive disease based on histopathological evidence of tissue invasion by fungi. Invasive diseases are divided into three categories, namely: acute invasive (fulminant), granulomatous invasive, and chronic invasive. Acute invasive FRS is defined as infection occurring <4 weeks with predominant vascular invasion occurring in immunocompromised patients. *Aspergillus* species, or member of zygomycetes is the most common cause. Granulomatous invasive FRS is a fungal infection occurring> 12 weeks characterized by enlarging mass in the cheek, orbit, nose, and paranasal sinuses in immunocompetent host. *Aspergillus flavus* is the main etiological agent. Chronic invasive FRS is a fungal infection that occurs slowly and commonly affects the ethmoid and sphenoid sinuses. This type is often found in patients with acquired immunodeficiency syndrome (AIDS), diabetes mellitus, and corticosteroid treatment. *Aspergillus fumigatus* is the most common etiological agent [8]. Our case is an example of a type of acute invasive disease. Generally, the role of surgery in the management of IA is the removal of infected or necrotic tissue to avoid further morbidity and prevent mortality in order to improve the patient's functional and aesthetic outcomes. Nevertheless, the specific surgical treatments have varied broadly among several surgeons due to the wide-ranging clinical settings in each case. Here, we report one case of invasive maxillary aspergillosis in a patient with SLE. This work has been reported in line with the SCARE 2018 criteria [9].

## 2 Presentation of case

We report a case of a 22-year-old female with a chief complaint of having a hole in the roof of her mouth. More than ten years ago in 2009, she was diagnosed with SLE. Before that time, she was constantly having a fever of unknown origin, joint pain, malar rash, and butterfly rash. Following her complaints, she was then tested for anti-double stranded DNA (anti-dsDNA), antinuclear antibodies (ANA), and the results were high, suggesting that she had SLE. She was routinely treated by a rheumatologist and regularly given oral methylprednisolone 0.5 mg/kg/day. In 2018, she experienced worsened symptoms, shortness of breath, and was referred to our hospital and was hospitalized for a couple of days before being discharged.

During treatment at home, she complained of swelling and bleeding of her gums. She also reported her some of her teeth became loose and fell out, followed by the gums peeling down and developing a hole in the roof of her mouth involving the upper jaw bone.

**Fig. 1:**
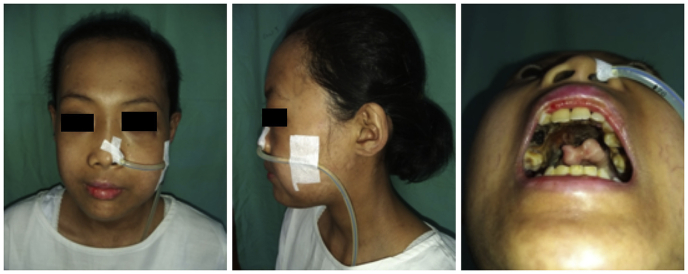
Preoperative view showing necrotic maxilla and palate perforation.

Afterward, she went to the Internal Medicine Department and Oral and Maxillofacial Surgery Department in our hospital. Perforation of the palate was seen in the examination (Fig. 1). However, no prompt action was given regarding the intraoral symptoms since the tissue was already necrotic. She was then treated with oral cyclophosphamide 125 mg/day, oral mycophenolate sodium 180 mg twice a day, and oral fluconazole 5 mg/day. The rheumatologist also asked her to stop the use of methylprednisolone.

In 2019, the palate (maxillary bone) excision biopsy was performed by an ear, nose, and throat (ENT specialist) doctor and an oral surgeon. The results revealed *Aspergillus* spp. invading the maxillary bone, and since then, she has been treated by a tropical medicine doctor before being referred to our Department of Plastic Reconstructive and Aesthetic Surgery for defect closure.

The findings of an axial computed tomography (CT) scan identified multiple osteo-destructions of the bilateral maxillary bones, bilateral zygomatic bones, right frontal bone, bilateral temporal bones, and right sphenoid bone.

**Fig. 2:**
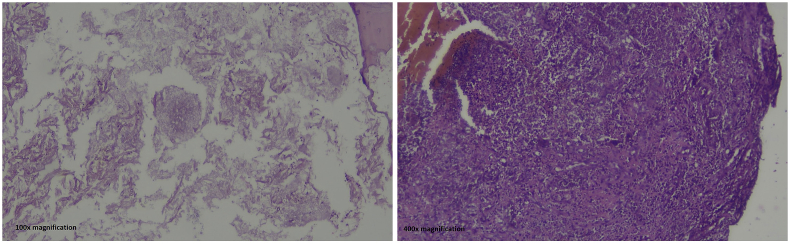
Patient rehabilitated with obturator which improves her quality of life.

The diagnosis of aspergillosis was established from the histological evidence of isolated maxillary bone collected from excision biopsy and stained using Periodic Acid-Schiff (PAS) showing fragments of bone tissues with degenerated epithelial and connective tissues infiltrated by abundant septate fungal hyphae and eosinophils (Fig. 2).

Thus, we assessed the patient with palatal (maxillary bone) perforation due to the invasive aspergillosis.

**Fig. 3:**
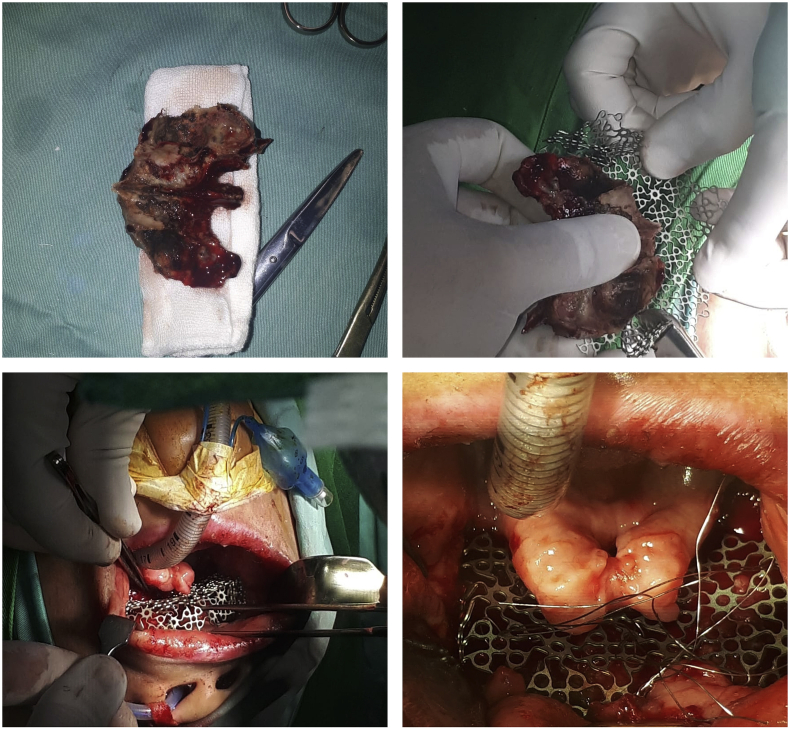
Excision biopsy of the maxillary bone revealed fungal components with tissue invasion and granulomatous reactions (PAS stain of maxillary bone at 100x and 400× magnification).

Multiple tooth (teeth) extractions followed by necrotomy of the palate (maxillectomy) and maxillary reconstruction were performed as the surgical management of the patient. During the procedures, these following teeth were extracted: 18, 17, 16, 12, 11, 21, 22, 23, 24, 26, 27, and 28. Following the tooth extractions, alveolectomy of the superior maxilla was performed using a bur freezer for further dental appliances. Necrotic maxilla was evident with no viable blood vessels nor soft tissues. Accordingly, we decided to perform necrotomy (maxillectomy) respecting the facial aesthetic subunits. The reconstruction of the maxillary bone (palate) was performed using two titanium mesh plates and screws (1.6 cm × 5 cm). Maxillary or palatal molding was done to create the obturator (prosthesis) for the patient in order to close off the palatal defect (Fig. 3).

**Fig. 4:**
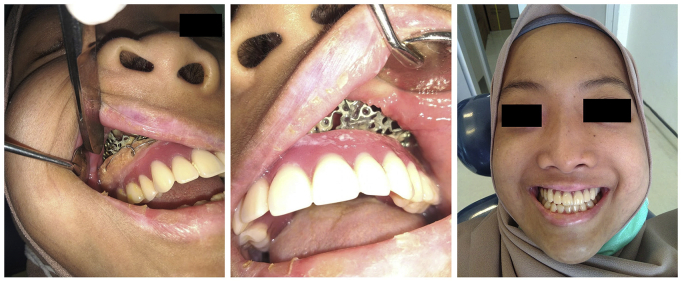
Intraoperative findings showing installation of obturator using titanium mesh.

The patient was successfully rehabilitated and followed-up routinely. There was no recurrence of the defect. Neither hypernasality or hyponasality were observed in the voice assessments, and neither regurgitation, aspiration, nor fistula were observed in the swallowing tests. There were no any other complications in the follow-ups. The patient was satisfied with the results (Fig. 4).

## 3 Discussion

IA of the soft tissue, particularly in the maxillary bone, is rare and classically affects immunocompromised patients such as those with SLE. However, the chronic disease caused by tissue invasion is unusual for the immunocompetent host [10,11]. Conversely, when the host is immunocompromised, the diagnosis of fungal infection should be considered. Prognosis of IA depends on the site of infection, extent of disease, and host factors. Patients with IA have mortality of nearly 100% when left untreated [12].

Halliday et al. also reported a quite similar case of invasive sino-orbital Aspergillus fumigatus infection occurred in immunocompromised patient. Old male presented with a 3-week history of painful left upper eyelid swelling. His patient underwent exenteration and received intravenous amphotericin B and voriconazole. The patient was died 3 months after initial presentation due to intracranial abscess, despite the treatment [13].

Ciecko et al. also reported a rare case of invasive fungal infection. He presented an old woman with complaint of left-sided purulent rhinorrhea and a lump inside her left nasal vestibule. She was in immunocompromised status due to her history of double lung transplantation, and was receiving tacrolimus and prednisone as an anti-rejection regimen. *Paecilomyces lilacinus* infection was found from her nasal biopsies. Oral voriconazole treatment for 5 months and surgical debridement was performed. The outcome was good and no evidence of fungal infection on repeated biopsies [14]. These two cases are similar to ours, especially from the patient's immunocompromised status, use of antifungal agent, and surgical debridement.

Generally, the role of surgery in managing IA is the elimination of infected necrotic tissue to avoid the spreading of infection and to reduce morbidity and mortality. However, once the maxilla is involved, surgical resection and debridement of the infected areas can result in extensive maxillary defects creating a challenge for the surgeon to replace not only the avulsed teeth but also the loss of soft tissues and bones, involving alveolar and hard palate.

Multiple tooth extractions followed by necrotomy (maxillectomy) and maxillary reconstruction were performed for the patient. The patient was also rehabilitated and followed-up routinely following the surgery to improve her function and quality of life. There were no recurrence, fistula, nor any complications in the follow-ups. The patient was satisfied with both the functional and aesthetic results of the surgery.

Nevertheless, there is no consensus about particular surgical techniques or recommendations for IA since it depends on the clinical considerations such as host immunity and clinical manifestations, in each case.

CT scan and magnetic resonance imaging (MRI) can help to determine a diagnosis of IA. Bone erosion or destruction, opacification, soft tissue mass, and necrosis may be identified and are the classic CT scan findings of IA [15]. However, these findings are usually found late in the course of IA, suggesting the need for early surgical debridement to control the disease and patient survival.

A biopsy is essential to determine and confirm the diagnosis. Particular stains, including PAS, Grocott-Gomori's Methenamine Silver (GMS) stains, and Galactomannan, can be used to confirm the diagnosis. Septate hyphae that are seen microscopically are typical and specific for *Aspergillus* spp. [2,16].

The patient was operated on for the reconstruction of the maxillary bone, and there was no evidence of fistula nor other complications in the follow-ups. The medical and surgical management were performed successfully. Additionally, the results show early therapy is important in efforts to improve the patient's outcome [17].

## 4 Conclusion

Invasive fungal infection is a potentially harmful disease, especially in immunocompromised patients. The possibility of fungal infection should be kept in mind, especially if we found bony erosion or necrosis in immunocompromised patient. The management of invasive fungal infection can vary from pharmacological therapy to control any underlying diseases, to surgical intervention such as debridement and necrotomy or amputation for necrotic tissues. Furthermore, antifungal therapy and prompt.

## Consent of patient

Written informed consent was obtained from the patient for publication of this case report and accompanying images. A copy of the written consent is available for review by the Editor-in-Chief of this journal on request.

## Provenance and peer review

Not commissioned, externally peer reviewed.

## Funding

The authors declare that this study had no funding source.

## Ethical approval

The informed consent form was declared that patient data or samples will be used for educational or research purposes. Our institutional review board also do not provide an ethical approval in the form of case report.

## Guarantor

Ishandono Dachlan

## Author contribution

Ishandono Dachlan conceived the study and approved the final draft. Aditya Rifqi Fauzi, Aditya Wicaksana drafted the manuscript. Siti Isya Wahdini, Muhammad Rosadi Seswandhana, Nurardhilah Vityadewi, Muhammad Bakhrul Lutfianto, and Franciscus Wihan Pradana critically revised the manuscript for important intellectual content. All authors read and approved the final draft. All authors facilitated all project-related tasks.

## Declaration of competing interest

No potential conflict of interest relevant to this article was reported.
